# Reactive Conductive Ink Capable of In Situ and Rapid Synthesis of Conductive Patterns Suitable for Inkjet Printing

**DOI:** 10.3390/molecules24193548

**Published:** 2019-09-30

**Authors:** Yuehui Wang, Dexi Du, Zhimin Zhou, Hui Xie, Jingze Li, Yuzhen Zhao

**Affiliations:** 1 Zhongshan Institute, University of Electronic Science and Technology of China, Zhongshan 528402, China; dudexi_work@foxmail.com (D.D.); huixiefly@126.com (H.X.); 2 School of Materials and Energy, University of Electronic Science and Technology of China, Chengdu 610054, China; lijingze@uestc.edu.cn; 3 Department of Materials Science and Engineering, Tsinghua University, Beijing 100084, China; zhaoyz@mail.tsinghua.edu.cn

**Keywords:** reactive ink, silver nanoparticles, inkjet printing, conductive pattern

## Abstract

We report a fabrication method of the conductive pattern based on in situ reactive silver precursor inks by inkjet printing. The reactive silver precursor inks were prepared with ethylene glycol and deionized water mixture as the solvent, and silver nitrate as silver source. Sodium borohydride solution as the reducing agent was first coated on photographic paper by screen printing process, and then dried at 50 °C for 4 h. Furthermore, the reactive silver precursor inks were printed on a photographic paper coated with sodium borohydride using inkjet printing to form silver nanoparticles in situ due to redox reaction, and thus a conductive pattern was obtained. The effects of the reactive silver precursor ink concentration and printing layer number and treatment temperature on the electrical properties and microstructures of the printed patterns were investigated systematically. The size range of in situ-formed silver nanoparticles was 50–90 nm. When the reactive silver precursor ink concentration was 0.13 g/mL, the five-layer printed pattern exhibited a sheet resistance of 4.6 Ω/γ after drying at room temperature for 2 h; furthermore, the sheet resistance of the printed pattern decreased to 1.4 Ω/γ after drying at 130 °C for 2 h. In addition, the display function circuit was printed on the photographic paper to realize the display of the numbers 0–99. It provides new research ideas for the development of environmentally friendly and low-cost flexible paper-based circuits.

## 1. Introduction

With the development of flexible electronics, printing electronics have shown a low-cost, environmentally friendly manufacturing process and excellent performance that is lightweight, flexible, and foldable, and which increases device integration density and reduces parasitic capacitance [1,2]. Inkjet printing technology in the printing of electronic manufacturing has aroused great interest in inkjet printing sensors [3,4], super capacitors [5,6,7], transistors [8,9], organic light emitting diodes [10,11], radio-frequency identification [12,13], and other forms of technology. Conductive inks are among the important components of inkjet printing. Conductive ink has a great influence on the quality of the printed pattern. Walker [14] proposed that a suitable conductive ink should have four requirements: a simple and high-yielding synthesis method, a moderately low viscosity, excellent electrical conductivity, and good stability at room temperature. Generally, it is challenging to obtain a high-performance inkjet conductive ink with both high stability and high electrical conductivity.

In recent years, significant efforts and progress has been made in developing printable silver inks [15,16,17,18,19,20,21,22]. Nanoparticle suspension and organometallic compounds are the two primary precursors to this. For silver nanoparticle conductive ink, organic stabilizers are required to create a stable silver nanoparticle suspension, and relatively high temperature annealing is also needed after printing to remove organic stabilizers and improve the conductivity [1,14,23,24]. However, nanoparticle-based conductive inks easily result in the blockage/clogging of inkjet printer nozzles because of agglomeration and settlement of nanoparticles [1,14]. Particle-free organometallic compound ink can avoid nozzle clogging, but requires high heat treatment in order for metalorganic decomposition to form conductive nanoparticles [25,26,27,28]. Moreover, there are limitations on the solubility of the silver source in the solvent and the choice of the decomposition temperature [26,27,28]. In addition, because of the poor adhesion between nanoparticles and the substrate, nanoparticles in conductive patterns described above are easily peeled off or can crack in the application process [29].

To address these issues, particle-free in situ reactive inks have been recently developed, with great advantages in their simple, low-cost fabrication process and high conductivity using relatively low treatment temperature [30,31,32,33,34,35,36,37,38]. Bidoki et al. reported that deposited layers of in situ reactive silver inks exhibited high electrical conductance of up to 1.89 × 10^5^ S/m [30]. Most recently, Lei et al. demonstrated that microscale-conductive silver features can be fabricated on the basis of in situ reactive silver inks on various flexible substrate at 90 °C [32]. On the basis of reactive silver inks, different printing methods such as electrohydrodynamic printing [32], microreactor-assisted printing [34], and inkjet printing [30,34,35,36,37,38] have been used to produce highly conductive silver features at relatively low temperatures. However, there are still areas for improvement.

In this paper, we report a fabrication method of the conductive pattern based on in situ reactive silver inks by inkjet printing. The in-site reactive silver inks were prepared with ethylene glycol and water mixture as the solvent, and silver nitrate as silver source. Sodium borohydride solution as reductant was first coated on photographic paper by screen printing process, and then the reactive silver inks were printed on a photographic paper coated with sodium borohydride using inkjet printing to form silver nanoparticles in situ because of redox reaction, and thus conductive patterns were obtained. Furthermore, the effects of the reactive ink concentration, printing layer number, and treatment temperature on the electrical properties and microstructures of the printed patterns were investigated systematically. Compared with the literature reports [28,30,31,32,33,34], the advantages of our work are that it does not require special experimental setups. The conductivity of the conductive pattern could be controlled easily by changing the concentrations of the reductant and the reaction ink. Meanwhile, the reducing agents were printed on the surface of the photographic paper by screen printing, which not only generated the uniform distribution of the reducing agent on the designed pattern, but also penetrated into honeycomb-like structures of the photographic paper. After inkjet printing, silver nanoparticles formed in situ were distributed in the surface and penetrated into honeycomb-like structures of the photographic paper, and then formed more conductive channels and improved the adhesion between silver nanoparticles and the substrate. In addition, ethylene glycol as solvent and moisturizer helped prevent nozzle clogging and maintained a certain viscosity without additives. This method provides new research ideas for the development of environmentally friendly and low-cost flexible paper-based circuits.

## 2. Results and Discussion

**Figure 1:**
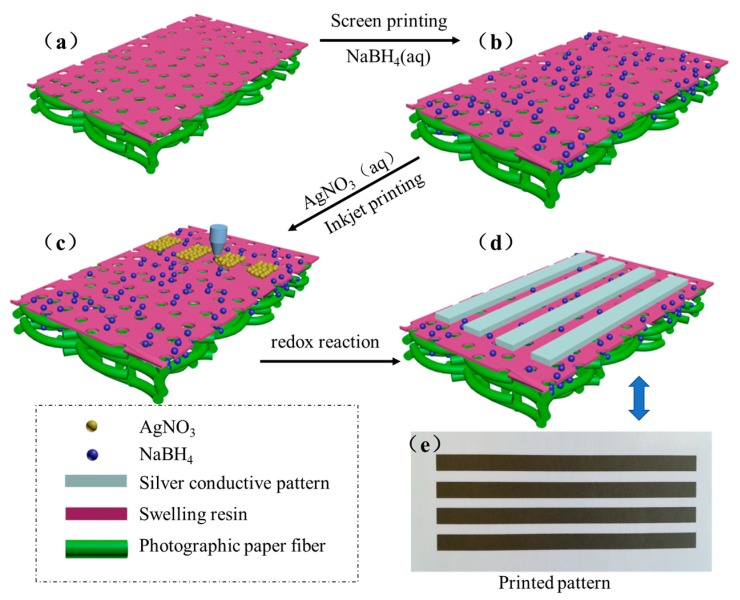
Schematic diagram of the process of the printable conductive patterns based on in situ reactive silver inks on the surface of the photographic paper. (**a**) Photographic paper, (**b**) NaBH_4_ coated on the surface of the photographic, (**c**) inkjet printing the reactive silver precursor on a photographic paper coated with sodium borohydride, (**d**) obtaining conductive patterns, (**e**) Printed pattern after dried.

Figure 1 shows a schematic of the process of the printable conductive patterns based on in situ reactive silver inks on the surface of the photographic paper. The resin-coat photographic paper is also called gap photographic paper or microporous photographic paper. Both of the sides of the paper were coated with waterproof polythene which contained the nanoscale silicon dioxide. The inorganic–organic composite coating had a honeycomb-like structure, and when sodium borohydride solution was coated on the paper by the screen printing process, it was quickly absorbed by the honeycomb-like micropores and attached to the paper. During inkjet printing, silver nitrate inks were sprayed to form a surface, and penetrated further into the photographic paper and reacted with the reducing agent. The reduction of AgNO_3_ by NaBH_4_ can be formulated as follows:

AgNO_3_ + NaBH_4_ + 3H_2_O = Ag + NaNO_3_ + B(OH)_3_ + 3.5 H_2_ (1)

With the increase of printing cycles, more and more silver nanoparticles formed in situ, covering the surface of the photographic paper, and then the printed pattern became fabricated.

**Figure 2:**
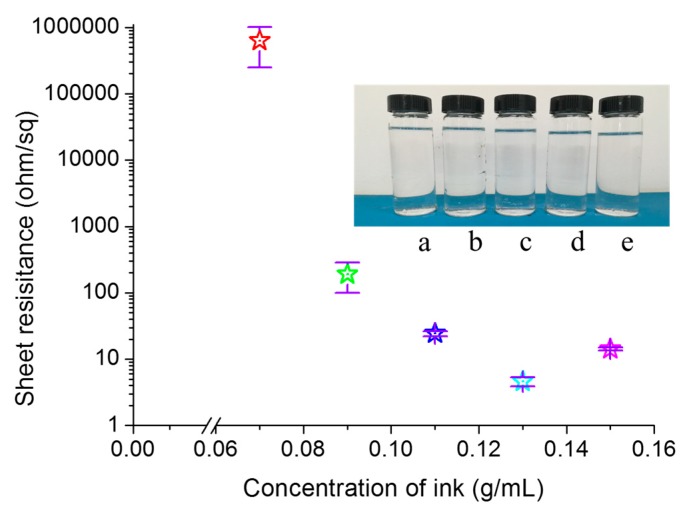
Sheet resistance of the printed patterns fabricated with different concentrations of the reactive ink. The inset are photos of the reactive ink with the concentrations of (**a**) 0.07, (**b**) 0.09, (**c**) 0.11, (**d**) 0.13, and (**e**) 0.15 g/mL.

Figure 2 illustrates the relationship between the sheet resistance of printed pattern and the reactive ink concentration. The insets in Figure 2 are photos of samples with the reactive ink concentrations of 0.07, 0.09, 0.11, 0.13, and 0.15 g/mL from (a) to (e), respectively. The silver nitrate solution had good stability and remained clear after over 30 days at room temperature. The conductive pattern was printed on 5 layers with the reactive ink concentrations of 0.07, 0.09, 0.11, 0.13, and 0.15 g/mL for each layer. The samples were dried at room temperature without any post-processing. As shown in Figure 2, the sheet resistance of the conductive patterns first decreased then increased as the concentration of the reactive ink increased. As the reactive ink concentration increased from 0.07 to 0.13 g/mL, the sheet resistance of the corresponding pattern decreased shapely from 630,600 to 4.6 Ω/γ. When the reactive ink concentration was 0.15 g/mL, the sheet resistance of the printed pattern increased to 14.26 Ω/γ, which was 4.6 times that of the printed pattern fabricated with the reactive ink concentration of 0.13 g/mL. The reason may be that the high concentration caused the reaction rate to be too fast and thus a large amount of gas was released, which led to the decrease of the compactness of the silver nanoparticles formed in situ, and thus the sheet resistance of the printed pattern increased.

**Figure 3:**
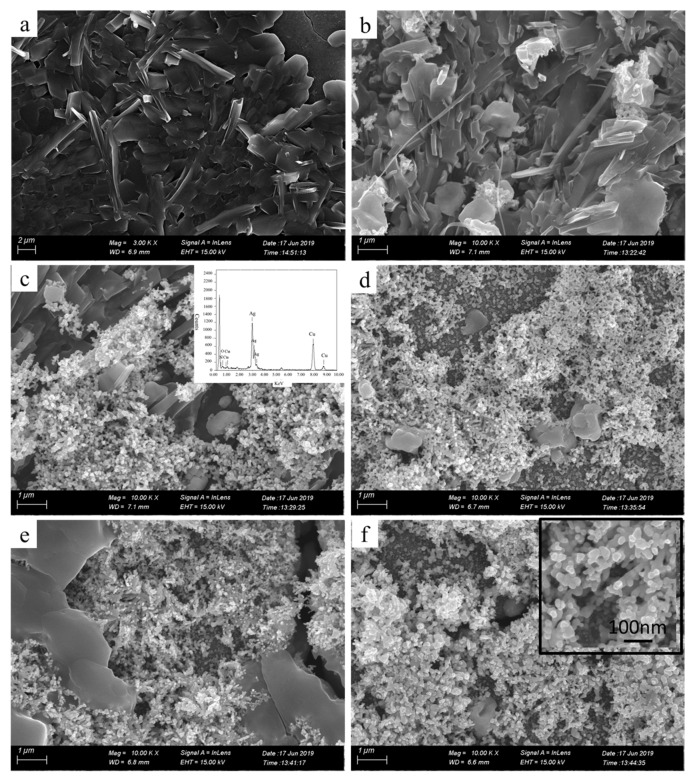
SEM images of the printed patterns fabricated with the reactive ink concentrations: (**a**) pure photographic paper, (**b**) 0.07, (**c**) 0.09, (**d**) 0.11, (**e**) 0.13, (**f**) 0.15 g/mL. The insets in (**c**,**f**) are the energy dispersive spectrometer (EDS) image and the local magnification image, respectively.

**Figure 4:**
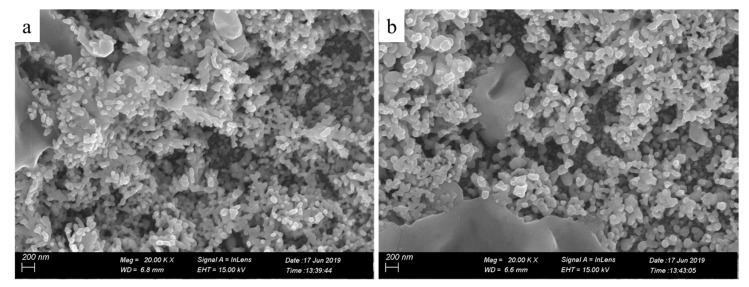
High magnification SEM images of the printed patterns with the reactive ink concentrations: (**a**) 0.11, (**b**) 0.15 g/mL.

To further characterize the printed patterns, we assessed microstructures of the printed patterns by SEM (Figure 3). The insets in Figure 3c,f are the energy dispersive spectrometer (EDS) image and the local magnification image, respectively. Seen from Figure 3a, the pure photographic paper was a microporous structure comprising interwoven fibers. After coated sodium borohydride solution, there was no obvious change in the surface structure of the pure photographic paper. Meanwhile, sodium borohydride was deposited on the surface of the photographic paper and penetrated into the honeycomb-like structure. Seen from Figure 3b–f, the silver nanoparticles formed in situ coated on the surface and deposited on the fiber of the photographic paper. Silver nanoparticles were joined together to form clusters with sintered-like structure [17], as shown in Figure 4. The size range of in situ formed nanoparticles was 50–90 nm. It is clearly visible that as the reactive ink concentration increased, the silver nanoparticles formed in situ gradually increased; thus, the contact area between nanoparticles increased, which prompted the decrease of the sheet resistance of the printed pattern. It is obvious that the inkjet printing process is capable of formatting silver nanoparticles on the photographic paper because of the reduction of silver ions to metallic silver.

**Figure 5:**
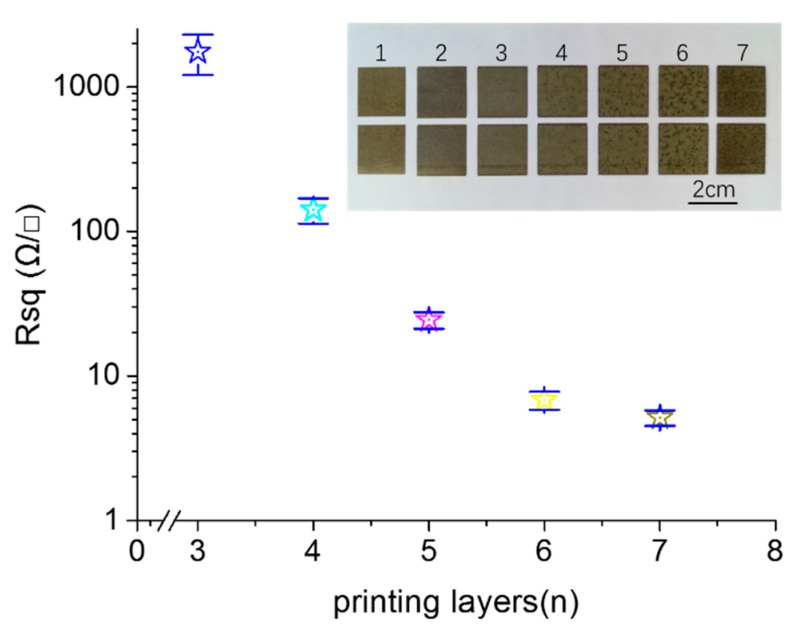
Sheet resistance of the printed patterns with different layers. The inserts are photos of the samples.

In order to further explore the effect of the microstructures on the electrical properties of the printed pattern, we fabricated the printed patterns with different printed layers. The reactive ink concentration was 0.11 g/mL. Figure 5 shows the sheet resistance of the printed patterns fabricated with the printed layers from 1 to 7. The insets in Figure 5 are the photos of the corresponding samples. When the number of printing layers was less than three, the printed pattern was non-conductive. The reason behind this is that there were too few silver nanoparticles formed in situ to connect with each other to form an effective conductive path. When the printed layers were three in number, the sheet resistance of the printed pattern was 1750 Ω/γ, indicating that the amount of silver nanoparticles formed in situ exceeded the percolation threshold, wherein the effective conductive paths were formed [21,27]. In increasing of the number of printing layers, the sheet resistance of the printed pattern gradually decreased. When the number of printing layers was six and seven, the sheet resistances of the printed patterns were 6.82 and 5.15 Ω/γ, respectively, indicating that many of the effective conductive paths were established for the printed pattern with six layers, and that after that, there were little effects the layers on the electrical resistivity. The electrical conductivity of the printed pattern was achieved by contact between the silver nanoparticles formed in situ. With the increasing of the printing layer, more silver nanoparticles were formed, which increased the contact area between nanoparticles and improved the electrical conductivity [26]. We can see from the photos of the samples that the color of the photos deepened as the number of printing layers increased. This was caused by increasing in the amount of the silver nanoparticles formed in situ.

**Figure 6:**
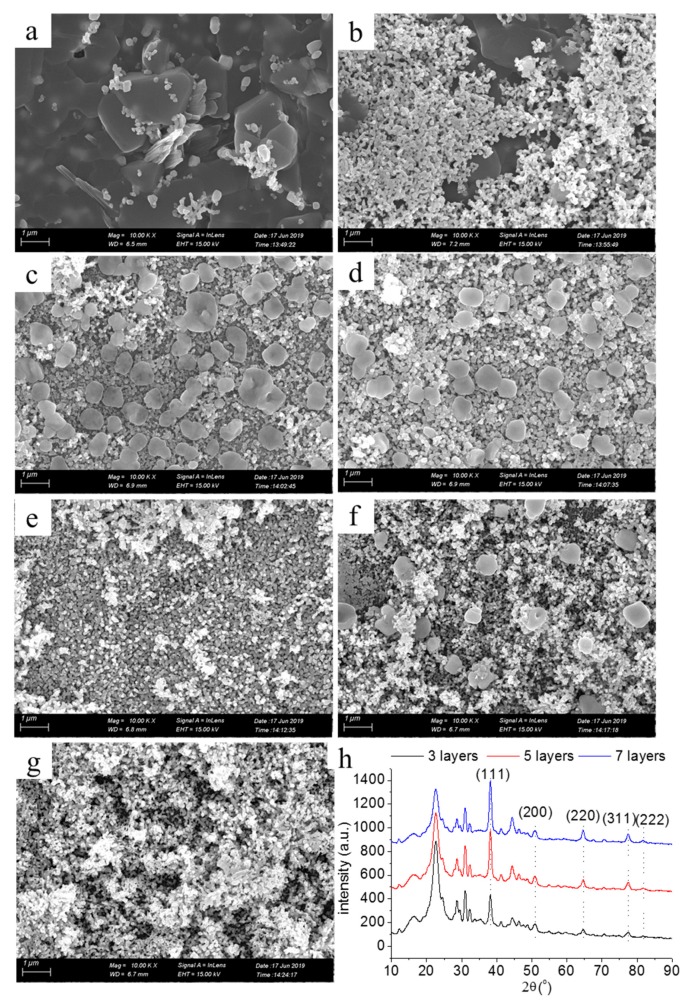
SEM images of the printed patterns with printing layers numbering (**a**) one, (**b**) two, (**c**) three, (**d**) four, (**e**) five, (**f**) six, (**g**) seven; and (**h**) XRD.

Figure 6a–g shows SEM images of the printed patterns with one to seven layers. The coverage of the surface of the photographic paper by silver nanoparticles was poor, and the exposure of fibers was evident when the printing layer was one (Figure 6a). The higher the number of printing layers, the more silver nanoparticles formed in situ, deposited on the surface of the photographic paper (Figure 6b–g). After seven layers, the surface of the photographic paper was completely covered with silver nanoparticles. Figure 6h shows the XRD pattern of the printed patterns with layers numbering three, five, and seven. Five distinct diffraction peaks in the XRD patterns can be seen: 2θ was 8.1°, 44.3°, 65.5°, 77.4°, and 81.6°, and the corresponding diffraction planes were (111), (200), (220), (311), and (222), respectively, which identify that the silver nanoparticles had face-centered cubic crystal structures [26].

**Figure 7:**
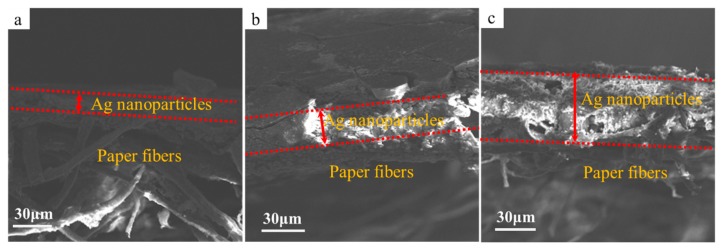
SEM images of the cross-section of the printed patterns with printing layers numbering one (**a**), three (**b**), and five (**c**).

Figure 7 shows SEM images of the cross-section of the printed patterns fabricated with printing layers numbering one, three, and five. Seen from Figure 7a, the silver nanoparticles formed in situ were deposited on the surface of the photographic paper after being printed once, and the thickness of the layer was about 12.19 μm. As the printing layers increased to three and five layers, the deposition thickness of the printed pattern increased to 30.96 μm (Figure 7b) and 65.39 μm (Figure 7c), respectively.

**Figure 8:**
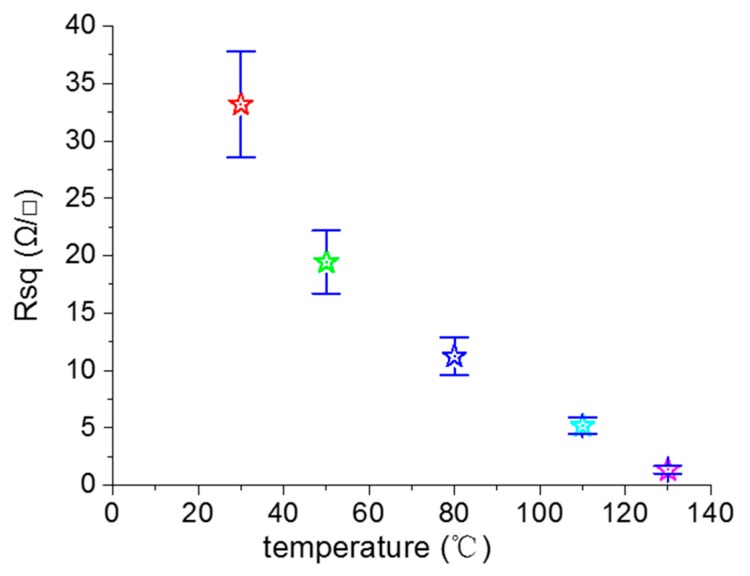
Relationship between the sheet resistance of the printed pattern and the heat treatment temperature.

We next investigated the effect of the heat treatment temperature on the electrical properties of the printed pattern. We fabricated the four-layer printed patterns by using 0.11 g/mL reactive inks that were treated at 30, 50, 80, 110, and 130 °C for 2 h. Figure 8 shows the relationship between the sheet resistance of the printed pattern and the heat treatment temperature. As the temperature further increased from 30 °C to 130 °C, the sheet resistance of the printed pattern decreased from 33.2 to 1.4 Ω/γ. It is obvious that the heat treatment temperature had a slight effect on the sheet resistance of the sample. The reasons behind this were that the residual ethylene glycol can volatilize at high temperatures, and the contact between the silver nanoparticles formed in situ were closer at high temperatures [37,38]. The electric conductivity of the printed pattern depended on the concentration of reactive ink, the printed layer number, the substrate, and the heat-treatment temperature, among other factors. We summarized some of the results reported in the literature, as shown in Table S1 (Supporting Information, Table S1).

**Figure 9:**
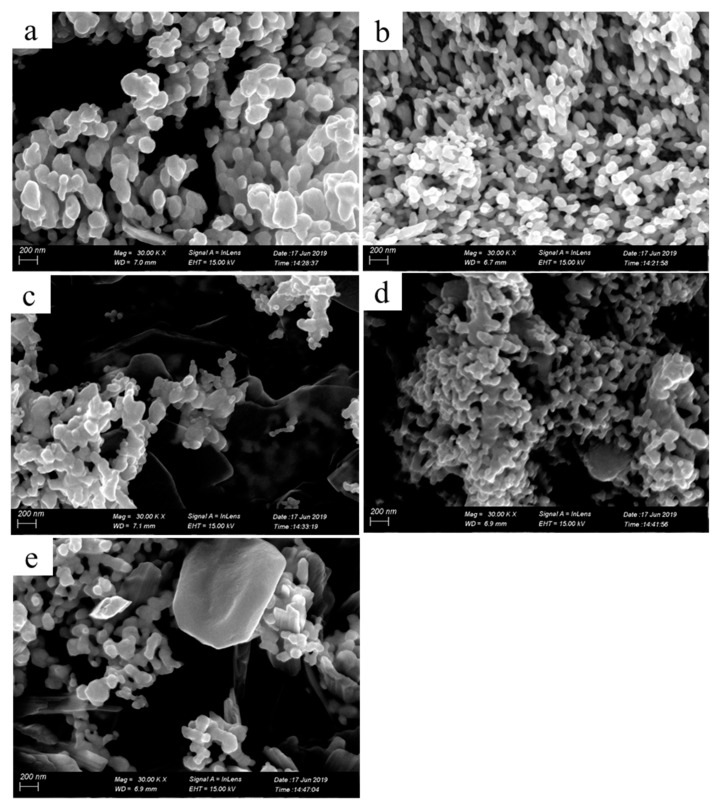
SEM images of the printed patterns with four printing layers treated at 30 (**a**), 50 (**b**), 80 (**c**), 110 (**d**), and 130 °C (**e**) for 2 h.

Figure 9 shows SEM images of the four-layer printed patterns fabricated with 0.11 g/mL reactive inks that were treated at 30, 50, 80, 110, and 130 °C for 2 h. Seen from Figure 9a–e, there were no significant differences in the microstructure of the samples. 

**Figure 10:**
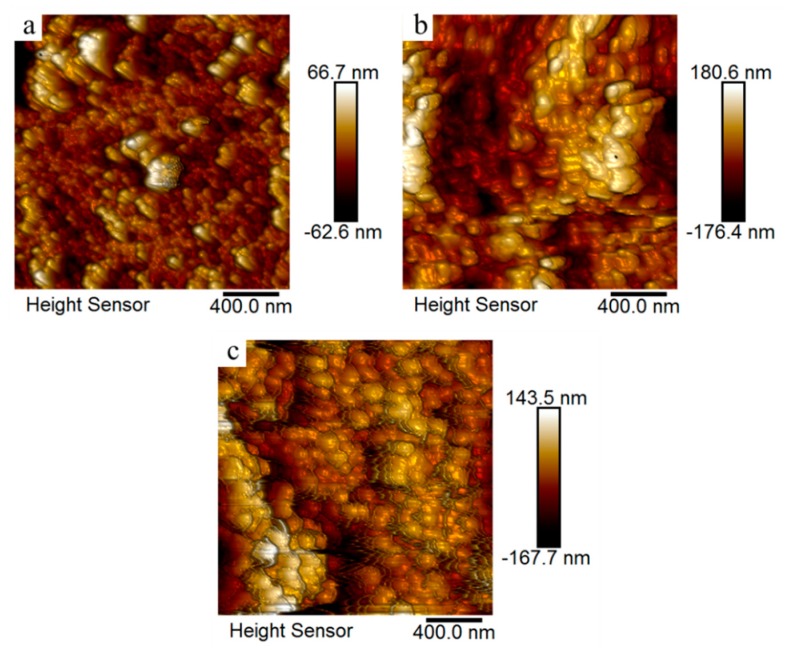
Atomic force microscopy images of the photographic paper coated with sodium borohydride (**a**) and the printed patterns treated at 30 (**b**) and 130 °C (**c**) for 20 min.

The surface topography of the photographic paper coated with sodium borohydride (Figure 10a) and the printed patterns treated at 30 (Figure 10b) and 130 °C (Figure 10c) were characterized using atomic force microscopy (AFM) operating in tapping mode. The measured root mean square (RMS) roughness values for the corresponding samples were 11.3, 21.9, and 27.4 nm, respectively; these values were all smaller than the size of the silver nanoparticles formed in situ. However, it is clear that the RMS value decreased along with the heat treatment temperature. The possible reason for this was that the sintering behavior of silver nanoparticles appeared at the temperature of the heat treatment and reduced the surface roughness.

**Figure 11:**
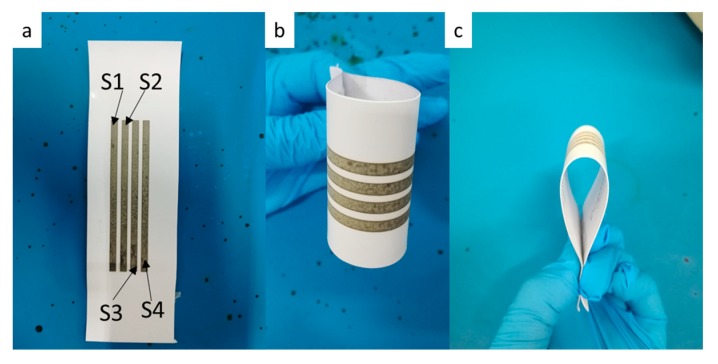
Photographs of the inkjet printing lines during bending cycles: (**a**) tiling, (**b**,**c**) 90° bending.

We fabricated four printed lines (3 × 80 mm) with five layers by using 0.11g/mL reactive ink, as shown in Figure 11, and further measured the change in the sheet resistance of the printed lines after bending cycles of 10, 100, 1000, 2000, and 3000, as shown Table 1. The bending cycles were carried out by 90° bending with both of ends of photograph (Figure 11). It can be seen that the relative change in sheet resistance of the printed pattern was slight, indicating that the printed pattern had good mechanical flexibility and stability.

**Figure 12:**
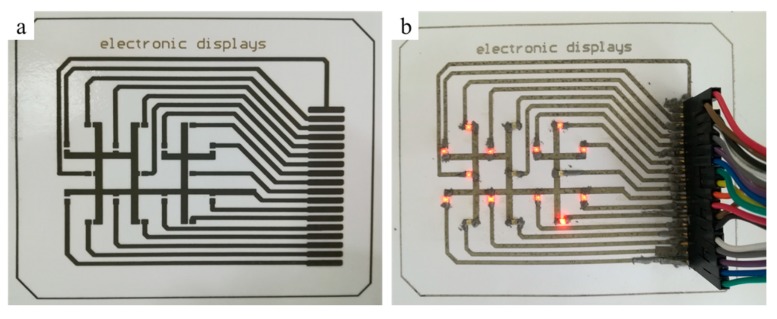
Inkjet printing digital display circuit (**a**) and digital display (**b**).

To demonstrate the applicability of the printed pattern, we fabricated the conductive circuits with the digital (0–99) display, as shown in Figure 12. The conductive circuits with four printing layers were fabricated by using 0.11g/mL reactive inks and treated at room temperature, and the patch of light emitting diode (LED) and row seat were welded using conductive adhesive at 130 °C. As shown in Figure 12, we succeeded in printing a simple electronic display on photographic paper, which can display the numbers 0 to 99, as shown in Figure 12 (see in the Supplementary Information, electronic displays.mp4). The digital display was composed of 17 patch LED beads with a power of 0.06 W, and the control signal and power supply were provided externally. The successful fabrication of the functional circuit proves the feasibility of the reactive ink and provides some ideas for future paper-based circuits.

## 3. Experimental Approach 

Silver nitrate (AgNO_3_, ≥99.8%) was purchased from Guangzhou Jinhuada Chemical Reagent Co., Ltd., Guangzhou, China. Sodium borohydride (NaBH_4_, AR), was purchased from Jinan Jiage Biological Technology Co., Ltd., Jinan, China, and ethylene glycol (AR) was purchased from Jinan Liyang Chemical Co., Ltd., Jinan, China. Resin-coat photographic papers (high gloss, 200 g/m^2^) were purchased from Foshan Guangshibo Office Supplies Co., Ltd., Foshan, China. All the chemicals were used as received. 

The different concentrations of reactive silver inks were obtained by dissolving different amounts of silver nitrate in the mixed solution of 3 mL deionized (DI) water and 27 mL ethylene glycol with stirring magnetically until completely dissolved. Then, the reactive inks were injected into the cartridge of the inkjet printer. Silver nitrate is attractive as a potential silver precursor ink because of its high water solubility and reasonable price. Ethylene glycol is a good solvent and moisturizer. By adding ethylene glycol into deionized water, the reactive ink viscosity can be adjusted to meet the requirements of inkjet printing and prevent nozzle clogging. The viscosities of all of the reactive silver inks were 10.01 mPa∙s, independent of the concentration of silver nitrate. In fact, we prepared the reactive inks with viscosities of 4.52, 8.29, 10.01, and 12.31 mPa∙s, separately, and studied the effect of ink viscosity on the inkjet printing process. The phenomenon of ink leakage was observed in the inkjet printing process when the viscosities of the inks were 4.52 and 8.29 mPa∙s. Meanwhile, discontinuous printing was observed when the viscosity of the ink was 12.31 mPa∙s, whereas good printing performance was observed when the viscosity of the ink was 10.01 mPa∙s. In this case, the surface tension of the ink was about 45.34 mN/m which was slightly lower than that of ethylene glycol.

The fabrication process of the conductive pattern was as follows: Sodium borohydride aqueous solution was prepared according to the stoichiometric ratio of sodium borohydride to silver nitrate of 1:1.1 and then was coated on the surface of the photographic paper by screen printing process, and then dried at 50 °C for 4 h. Furthermore, the reactive inks were printed on a photographic paper coated sodium borohydride using inkjet printing (HP DeskJet 1112 printer, Hewlett-Packard Co., Palo Alto, CA, United States) to form silver nanoparticles in situ due to redox reaction, and thus the conductive pattern was obtained. Note that sodium borohydride was excessive in order to ensure that silver nitrate could be fully reduced. The printed pattern was rinsed two to three times with deionized water (DI) water and was then quickly dried by using a hair dryer. The pattern was programmed as bitmap images. The printed conductive pattern was dried at room temperature or low temperature treatment until the solvent volatilized completely. We did not observe the clogging of the printheads under any of the experimental conditions.

The conductive circuits with the digital (0–99) display were fabricated by the five - layer printed designed patterns, and the printed conductive patterns were placed in room temperature or low temperature treatment, and the patch LED and row seat were welded with a low-temperature solder.

The microstructures of all of samples were characterized by scanning electron microscope (SEM; Zeiss Sigma 500, Carl Zeiss, Germany). Energy dispersive spectrometry (EDS) of samples were characterized by scanning electron microscope (SEM; Zeiss Sigma 500, Carl Zeiss, Germany). The sheet resistances of films were characterized using a four-probe system (ST2253, Suzhou Jingge Electronic Co., Ltd., Suzhou, China). The surface morphology was analyzed via atomic force microscopy (Dimension Edge, Bruker, Billerica, MA, United States), and five different areas of the surface of sample were selected to obtain root mean square roughness (RMS) value and were calculated as average value. The viscosity was measured by a viscometer (NDJ-1S, Shanghai Qili Scientific Instrument Co., Ltd., Shanghai, China). The surface tension of the ink was measured by automatic tension apparatus (JK99C, Shanghai Zhongchen Digital Technology Equipment Co., Ltd., Shanghai, China).

## 4. Conclusions

In summary, the in-site reactive silver precursor inks were prepared with ethylene glycol and water mixture as the solvent, silver nitrate as the silver source, and sodium borohydride as the reducing agent. Sodium borohydride solution was first coated on photographic paper by screen printing process, and then the reactive inks were printed on a photographic paper coated with sodium borohydride using inkjet printing to form silver nanoparticles in situ due to redox reaction, and thus conductive patterns were obtained after drying at room temperature. As the reactive ink concentration increased from 0.07 to 0.13 g/mL, the sheet resistance of the corresponding pattern decreased dramatically from 630,600 to 4.6 Ω/γ; when the reactive ink concentration was 0.15 g/mL, the sheet resistance of the printed pattern increased to 14.26 Ω/γ. The sheet resistance of the three-layer printed pattern was 1750 Ω/γ. When the number of printing layers were six and seven, the sheet resistances of the printed patterns were 6.82 and 5.15 Ω/γ, respectively. The heat treatment temperature had a slight effect on the sheet resistance of the sample because of the volatilization of the residual ethylene glycol and the close contacts between the silver nanoparticles formed in situ at high temperature. The bending test demonstrated that the printed pattern had good mechanical flexibility and stability. A simple function circuit was successfully printed, and can realize digital 0–99 display by using this method. Our work provides some research ideas for the development of paper-based circuits.

## Figures and Tables

**Table 1:** Resistance of the inkjet printing patterns after different bending cycles.

Bending Cycles (*n*)	S1 (ohm)	S2 (ohm)	S3 (ohm)	S4 (ohm)
0	41.8	34.3	36.3	41.8
10	41.9	34.4	39.7	43.1
100	41.3	34.5	41	43
1000	42.1	41.4	43.4	43.7
2000	45.1	41.6	47.6	44.5
3000	45.9	49.3	59.9	57.6

## Supplementary Materials

Supplementary Materials are available online.
