# Supplementary material for: Reactive Conductive Ink Capable of In Situ and Rapid Synthesis of Conductive Patterns Suitable for Inkjet Printing
Source: Molecules. 2019 Sep 30;24(19):3548. doi: 10.3390/molecules24193548 (PMC6803995; doi:10.3390/molecules24193548)
Supplement: Supplementary file 1 [file molecules-24-03548-s001.pdf]

Supporting Information

Reactive conductive ink capable of in-situ and rapid synthesis of conductive patterns suitable for inkjet printing

Dexi Du [1, 2], Yuehui Wang [1,*], Zhimin Zhou[1, 2], Hui Xie[1], Xianfeng Zhang[1,] Jingze Li[2,*], and Yuzhen Zhao[3]

[1] Zhongshan Institute, University of Electronic Science and Technology of China, Zhongshan, Guangdong Province, P. R. China; Post Code: 528402; dudexi_work@foxmail.com (D.D.); wangzsedu@126.com (Y.W.); huixiefly@126.com(H.X.); zhangxf07@gmail.com （X. Z.）,

[2] School of Materials and Energy, University of Electronic Science and Technology of China, Chengdu, Sichuan Province, P. R. China; Post Code: 610054; 1443908019@qq.com (Z. Z.); lijingze@uestc.edu.cn (L. Z)

[3] Department of Materials Science and Engineering, Tsinghua University, Beijing, P. R. China; Post Code: 100084; zhaoyz@mail.tsinghua.edu.cn (Y. Z.)

* Correspondence: wangzsedu@126.com; Tel.: +86-760-8832-5402; lijingze@uestc.edu.cn; +86-28-8320-7620

Table1 S1 Composition of the experimental conditions and electric conductivity of the printed pattern fabricated by reactive inks reported in literatures

Printing process	Ink composition	Printing layers (n)	Post treatment temperature	Sheet resistance	conductivity	Ref.
ink-jet printing	Reductant：ascorbic acid<br>Silver Source：$AgNO_3$	-	150 ℃	0.5 Ω/□	1.89 × 105 S·m^{-1}	30
inkjet printing	Reductant：$NaBH_4$<br>copper ink<br>copper Source：copper citrate<br>nickel ink<br>nickel Source：nickel sulfate	350/250	-	-	1.8 ×10^{6} S·m^{-1} (7.5um)/<br>2.2 × 10^{4} S·m^{-1} (7.5um)	31
Electrohydrodynamic printing	formic acid<br>Ammonium aqueous solution<br>Silver Source：Silver acetate<br>Ag–PEO inks	100	90 ℃	-	3.3 ×10^{6} S·m^{-1}	32
microreactor-assisted printing	Reductant：Formaldehyde<br>Silver Source：$Ag(NH_3)^{2+}$	-	RT	-	3.3 ×10^{7} S·m^{-1}<br>134 ±9 nm	33
inkjet printing	Silver Source：$AgNO_3$<br>Reductant：1-Dimethylamino-2-propanol	-	100 ℃	-	0.58±0.04×10^{5} S·m^{-1}（323.8 nm）	34
inkjet printing	Amepox MC NANO INK AX JP-60 n silver nanoink	8	130 ℃	0.62±0.03 Ω/□	-	35
inkjet printing	Commercially available inkjet-printable silver U5714	2	150 ℃	5.7 Ω/□	-	36
inkjet printing	Silver Source：$Ag(NH_3)_2CH_3CO_2$<br>Reductant：NH_4HCO_2	4	50 ℃	2.3 Ω/□	-	37
inkjet printing	organometallic reactive compounds<br>organometallic amine compound	8	140 ℃	0.2 ± 0.025 Ω/□	-	38
inkjet printing	Silver Source：$AgNO_3$<br>Reductant：$NaBH_4$	7/4	RT/130 ℃	5.15 /1.4 Ω/□	-	This work
